# Arrhythmic mitral valve prolapse in 2023: Evidence-based update

**DOI:** 10.3389/fcvm.2023.1130174

**Published:** 2023-04-18

**Authors:** Maciej Kubala, Benjamin Essayagh, Hector I. Michelena, Maurice Enriquez-Sarano, Christophe Tribouilloy

**Affiliations:** ^1^ Department of Cardiology, Amiens University Hospital, Amiens, France; ^2^ EA 7517, Jules Verne University of Picardie, Amiens, France; ^3^ Department of Cardiovascular Medicine, Mayo Clinic, Rochester, MN, United States; ^4^ Department of Echocardiography, Cardio X Clinic, France

**Keywords:** mitral valve prolapse (MPV), premature ventricular contractions, ventricular tachycardia (VT), sudden cardiac death (SCD), risk stratification

## Abstract

Patients with mitral valve prolapse (MVP) may develop ventricular arrhythmias, ranging from premature ventricular contractions through more complex non-sustained ventricular tachycardia to sustained life-threatening ventricular arrhythmias. The prevalence of MVP in autopsy series of young adults who died suddenly has been estimated to be between 4% and 7%. Thus, “arrhythmic MVP” has been reported as an underappreciated cause of sudden cardiac death, leading to a renewed interest in the study of this association. The term “arrhythmic MVP” refers to a small subset of patients who have, in the absence of any other arrhythmic substrate, MVP, with or without mitral annular disjunction, and frequent or complex ventricular arrhythmias. Our understanding of their coexistence in terms of contemporary management and prognosis is still incomplete. While literature regarding the arrhythmic MVP may be contrasting despite recent consensus document, the present review summarizes the relevant evidence concerning the diagnostic approach, prognostic implications, and targeted therapies for MVP-related ventricular arrhythmias. We also summarize recent data supporting left ventricular remodeling, which complicates the coexistence of MVP with ventricular arrhythmias. As the evidence for a putative link between MVP-associated ventricular arrhythmias and sudden cardiac death is scarce and based on scant and retrospective data, risk prediction remains a challenge. Thus, we aimed at listing potential risk factors from available seminal reports for further use in a more reliable prediction model that requires additional prospective data. Finally, we summarize evidence and guidelines on targeted therapies of ventricular arrhythmias in the setting of MVP, including implantable cardioverter defibrillators and catheter ablation. Our review highlights current knowledge gaps and provides an action plan for structured research on the pathophysiological genesis, diagnosis, prognostic impact, and optimal management of patients with arrhythmic MVP.

## Introduction

The association of mitral valve prolapse (MVP) with life-threatening ventricular arrhythmias (VA) has baffled cardiologists since the early 1980 s. There is currently a renewed interest in the study of this association, which has led to the development of new concepts. Mitral valve prolapse often presents with premature ventricular contractions (PVCs) (1) but only a small subset of patients with MVP are considered to be at high risk of malignant VA. “Malignant MVP” has been reported to be an underappreciated cause of sudden cardiac death (SCD) in young adults, as the prevalence of MVP in autopsy series has been estimated to be between 4% and 7% (2–4). The rate of SCD among patients with MVP has been prospectively estimated to range from 0.2% to 0.4% per year and to be 1.8% per year for patients with severe mitral regurgitation (MR) due to leaflet flail (5–7). Yet, the identification of subgroups of MVP patients with higher arrhythmic risk is still a challenge. This review focuses on current knowledge of clinical data, electrocardiographic aspects, and the specific imaging presentation of arrhythmic MVP, and summarizes evidence on risk stratification and management. In addition, the main knowledge gaps concerning diagnostic approaches and therapeutic options of arrhythmic MVP are highlighted.

### Clinical and ECG presentation

**Figure 1:**
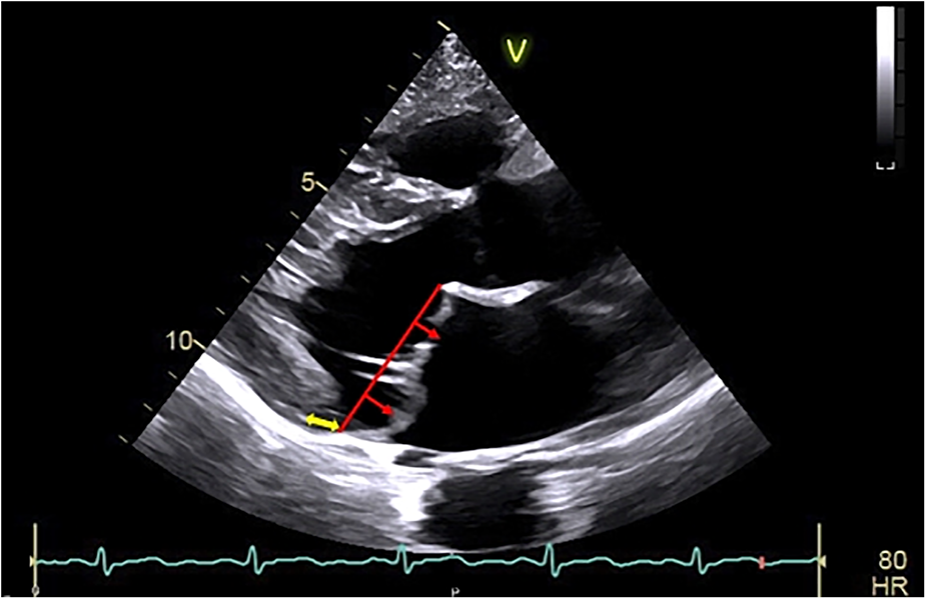
Arrhythmic mitral valve prolapse by transthoracic-echocardiography. Transthoracic-echocardiographic long-axis view in end-systole with a displacement of both leaflet >2 mm (red arrow) above the plane of the annulus (red line) defining a bileaflet mitral valve prolapse with thick leaflets linked to myxomatous degeneration. Note the presence of the detachment of the posterior leaflet from the left-ventricular myocardium (mitral annular disjunction, yellow arrow) to be assessed in dynamic analysis (that is a frame by frame analysis during the entire cardiac cycle) to better ascertain the position of the posterior mitral annulus.

Clinical presentation of the arrhythmic MVP may vary in severity, ranging from asymptomatic patients, through palpitations to presyncope/syncope (3). Syncope was shown to be a frequent preceding symptom for patients who developed cardiac arrest (8). While the initial focus was on bileaflet MVP as the main culprit, the arrhythmic MVP phenotype is multidimensional and characterized by severe myxomatous disease with marked leaflet redundancy and thickening, inferolateral mitral annular disjunction (MAD) (Figure 1) with frequent inverted T-waves in the inferior leads, frequent PVCs by ECG, more non-sustained ventricular tachycardia (VT) and a higher PVC burden by Holter monitoring than those with normal mitral valves (2, 8, 9). Phenotypic presentations of MVP with an increased risk of VA and/or SCD are shown in Table 1.

**Table 1:** Mitral valve prolapse phenotypes with increased risk of ventricular arrhythmias and/or sudden cardiac death.

		References
Clinical presentation	Syncope: 35% of MVP with malignant VAs or SCD	(8, 10)
Echocardiography	MR severity Severe MR: increased risk of SCD Reduced LV systolic function: LVEF ≤ 50%- increased risk of SCD Bileaflet myxomatous MVP: increased risk of VAs independent of MR severity MAD: increased risk of malignant Vas	(6) (6) (9) (9, 11)
Electrocardiography	T wave inversion in inferior and lateral leads: 65% of MVP with malignant Vas	(3, 8, 9)
Holter monitoring	Non-sustained VT runs ≥180 beats/min and/or history of sustained VT/VF: increased risk of mortality	(9)
Cardiac MRI	Replacement fibrosis in infero-basal wall and/or at the level of papillary muscle: increased risk of complex VAs	(3, 8, 10, 12, 13)
Genetics	Filamine C variant: increased risk of VAs	(14)

LV, left ventricle; MAD, mitral annular disjunction; MR, mitral regurgitation; MVP, mitral-valve prolapse; VF, ventricular fibrillation; VT, ventricular tachycardia.

**Figure 2:**
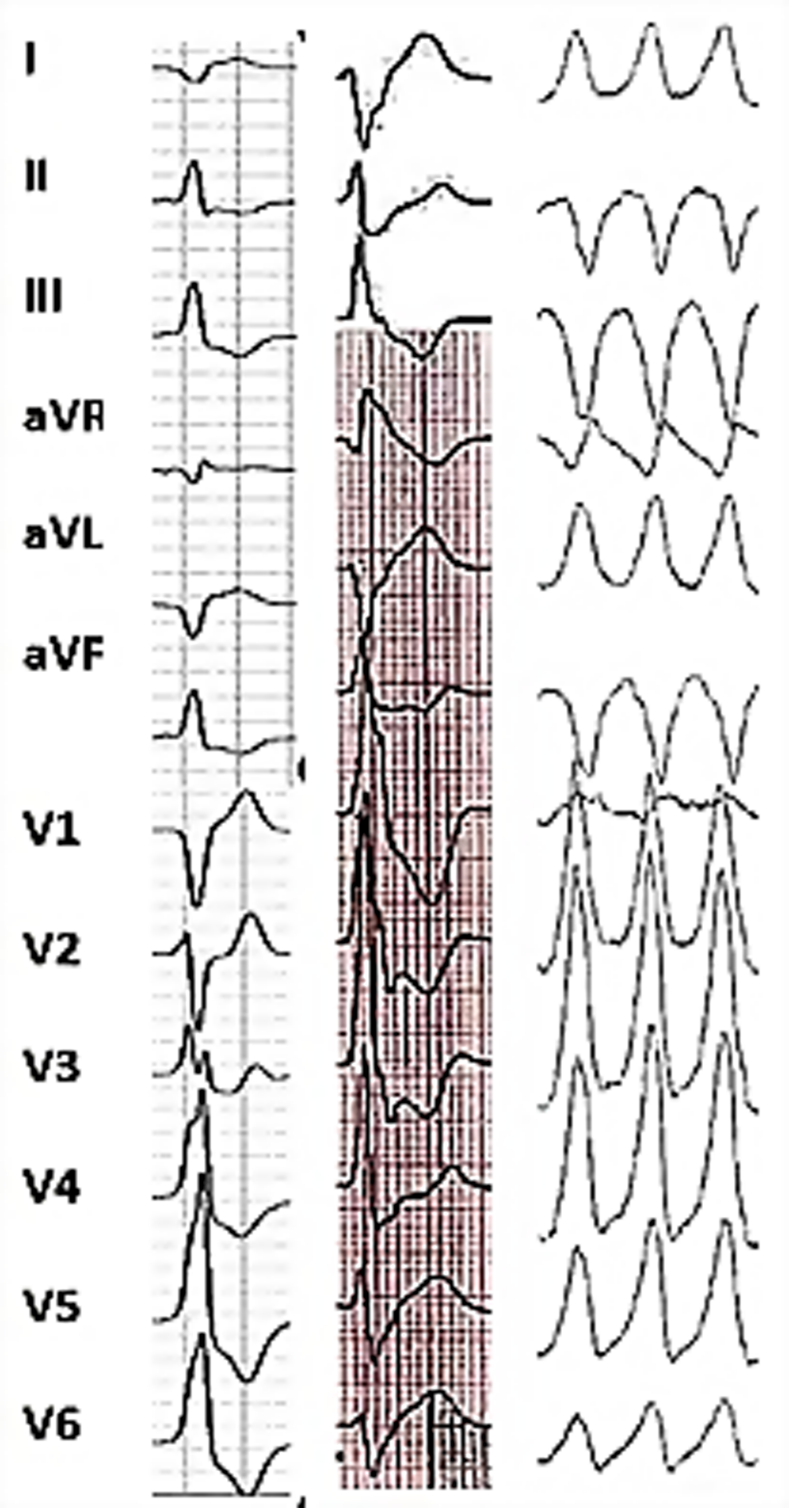
Representative 12-lead surface ECG illustrating multifocal ventricular arrhythmias in the same patient. Frequent premature ventricular complexes of 2 different morphologies were recorded: the first one showing left bundle branch block configuration, V3 transition and inferior axis consistent with an outflow tract site and the second one with right bundle branch block morphology, V5 transition and D2/D3 negative/positive discordance (meaning an opposite depolarization vector along bipolar limb leads II and III being equivalent to a frontal axis of +150 to +210°) suggesting the origin from the anterolateral papillary muscle. Sustained monomorphic ventricular tachycardia (on the right) with a right bundle branch block configuration and superior axis pointing towards the postero-basal left ventricular wall origin was induced with programmed electrical stimulation.

Early data showed a high prevalence of PVCs in MVP patients, ranging from 58% to 89% (1). A more recent prospective study on a large population of patients with different stages of MVP showed the utility of the exercise test to reveal PVCs and their potential site of origin for more than two-thirds of patients with MVP (15). Patients with identified isolated PVCs were older, presented more non-sustained VT during the exercise test, and more often had MR of stage >II, with “deeper” valve prolapse and longer MV leaflets. In this study, patients with PVCs also showed elevated T1 values for the postero-medial papillary muscle (PPM) and left ventricular infero-latero-basal wall by contrast-enhanced cardiac magnetic resonance (MRI) imaging. The most common PVC and non-sustained VT morphology was associated with the PPM. Other PVC morphologies suggestive of the mitral valve apparatus as the origin have been described in the setting of patients with arrhythmic MVP, including the antero-lateral papillary muscle, aorto-mitral continuity, or peri-mitral valve area, as well as more distant sites, such as the outflow tract, moderator band, or fascicular sites (Figure 2) (2, 15, 16).

### MRI findings

Cardiac MRI may help in the diagnosis of MAD as a part of the arrhythmic MVP complex as well as phenotypic markers of higher risk as fibrosis (17, 18). Indeed fibrosis localized to the PPM and left ventricular (LV) segments adjacent to the PPM, identified by MRI and in pathological studies, has been highlighted as a potential myocardial source of VA that complicates MVP (3, 10, 19, 20). A higher prevalence of replacement fibrosis in the LV infero-basal wall was identified in patients with MVP and complex VAs than those without VAs (73% vs. 7%) (3). In another study, LV fibrosis was not only associated with more frequent VAs but also with a more dilated LV and, interestingly, with MR severity, increasing from 13% of patients with mild MR to 37% of those with severe MR (12).

Contrast-enhanced cardiac magnetic resonance data of a large cohort of patients with primary MR showed that replacement fibrosis affected 36% of patients with MVP specifically in the basal segments, as well as extending to the mid inferolateral LV wall (10). In this study, the presence of replacement fibrosis was related to increased symptomatic ventricular arrhythmic events affecting 4.5% patients with MVP vs. 0.6% of patients in the non-MVP cohort. Furthermore, cases with MVP-SCD matched to control cases with noncardiac death showed increased LV anterior, lateral, posterior, and interventricular septum fibrosis, characterized by an endocardial-to-epicardial gradient (13). Left ventricular fibrosis was also found to be associated with systolic curling, described as an atypical motion of the posterior mitral ring (21).

### Left ventricular remodeling

The outcome in MVP is determined by both MR severity and its left-atrial and left-ventricular consequences (22, 23). Disproportionate LV dilation has been reported for 16% of patients without significant MR not exposed to significant volume overload, in addition to excess annular enlargement in systole, suggestive of an MVP-associated myocardial disease (12, 24). Larger LV dimensions were independent of the degree of replacement fibrosis but were linked to more frequent PVCs. A study by Yang et al. also showed that patients with less-than-moderate degenerative MR due to MVP exhibited early LV remodeling independent of MR progression but strongly associated with more frequent PVCs (25). The question of whether such myocardial changes are linked to the MVP phenotype itself or rather the consequence of PVC-induced cardiomyopathy remains unanswered.

### Sudden cardiac death

In previous studies, severe VAs were rarely documented before SCD (6), possibly due to the scarcity of Holter-monitoring performed. However, patients who present with VA by monitoring show progressive but significant excess mortality (9).

The yearly incidence of SCD in patients with MVP has been estimated to be 0.2%–0.4% and up to 1.8% per year for those with severe MR due to leaflet flail (5–7, 26, 27). Arrhythmic MVP without severe MR has been suggested to be an underestimated cause of SCD among young adults (2, 3). However, patients with MVP and proven VA are often in their 60 s (9). Severe VA, defined as non-sustained VT ≥ 180 beats/min or a proven history of sustained VT/VF, is infrequent (9%) but associated with excess mortality subsequent to its diagnosis (9).

Mitral annular disjunction in the context of MVP diagnosed by cardiac MRI or by standard transthoracic echocardiography after cardiac arrest (3, 8, 18) has been suggested to be a harbinger of SCD. The MAD-VA link is hypothesized to be subsequent to local fibrosis induced by the tension generated by such an ample prolapse (21). MVP patients with MAD developed within the first 10 years more frequent VA than those without MAD (11). However, recent outcomes data in MVP patients demonstrate that MAD may be associated with the secondary development of arrhythmias but is not associated with excess mortality during the first 10 years and should not, *per se*, lead to risky electrophysiological interventions (11). Of note, MAD is a common finding in the general population and only rare inferolateral MAD is associated with MVP and myxomatous MV disease (28). MVP is frequent and MAD is identified in up to 40% of patients with connective tissue diseases (29, 30). In patients with Marfan syndrome and Loeys–Dietz syndrome MAD was associated with increased risk of mitral surgery, but was not linked to higher risk of life-threatening VA (29). In addition, no association has been found between MAD and the PVC site of origin (15). Therefore, although MAD is a component of the multifaceted arrhythmic MVP phenotype, it does not appear by itself, to be a direct harbinger of SCD. Thus, in the presence of MAD careful monitoring is recommended and MAD should not, by itself, lead to risky electrophysiological interventions (17). Notably, some authors challenge the currently used definition of MAD based only on its systolic description that can be partly biased by including cases of juxtaposition of posterior leaflet and atrial wall (31). They identify a less prevalent “true MAD” defined as the insertion of the leaflet on the atrial wall observed in diastole with the presence of a separating sub-valvular membrane. These uncertainties could lead to false stratification of arrhythmic risk in MVP patients and need more clarification. Possible inheritable proarrhythmic genetic substrate in the form of cardiomyopathy-causative variants of FLNC, encoding filamin C, has been reported in patients with arrhythmic MVP (14).

Additional research is needed to establish the optimal approach for risk stratification for patients with MVP, including the prognostic role of cardiac MRI. Severity and the prognostic implication of arrhythmias in patients with MVP will require large registries to provide sufficient power to define clinical determinants and outcomes accounted for by well-defined covariates. The utility of treadmill tests and loop recorders with remote monitoring for stratification of the risk of SCD in arrhythmic MVP needs to be defined by prospective cohorts with long-term follow-up. Additional research is also needed to determine the precise role of advanced electrophysiological testing, including programmed ventricular stimulation in the setting of MVP associated VAs.

### Management of MVP with ventricular arrhythmia

Most cases of severe organic MR are treated by surgery. Surgical cryoablation for VA during cardiac surgery has been reported but data on long-term results are lacking (32, 33). Nonetheless, the correction of flail leaflet is possibly associated with a lower risk of SCD (6, 9) and some authors suggest that VA-excess mortality tends to disappear post MV surgery with MAD disappearance (9, 24). However, non-sustained VT by Holter-monitoring following MR repair or replacement may be a long-term predictor of SCD (34). After surgical MR repair or replacement, an implantable cardioverter-defibrillator (ICD) is indicated as a class I recommendation for patients who satisfy implantation criteria for secondary prevention of SCD according to current ESC Guidelines (35).

**Figure 3:**
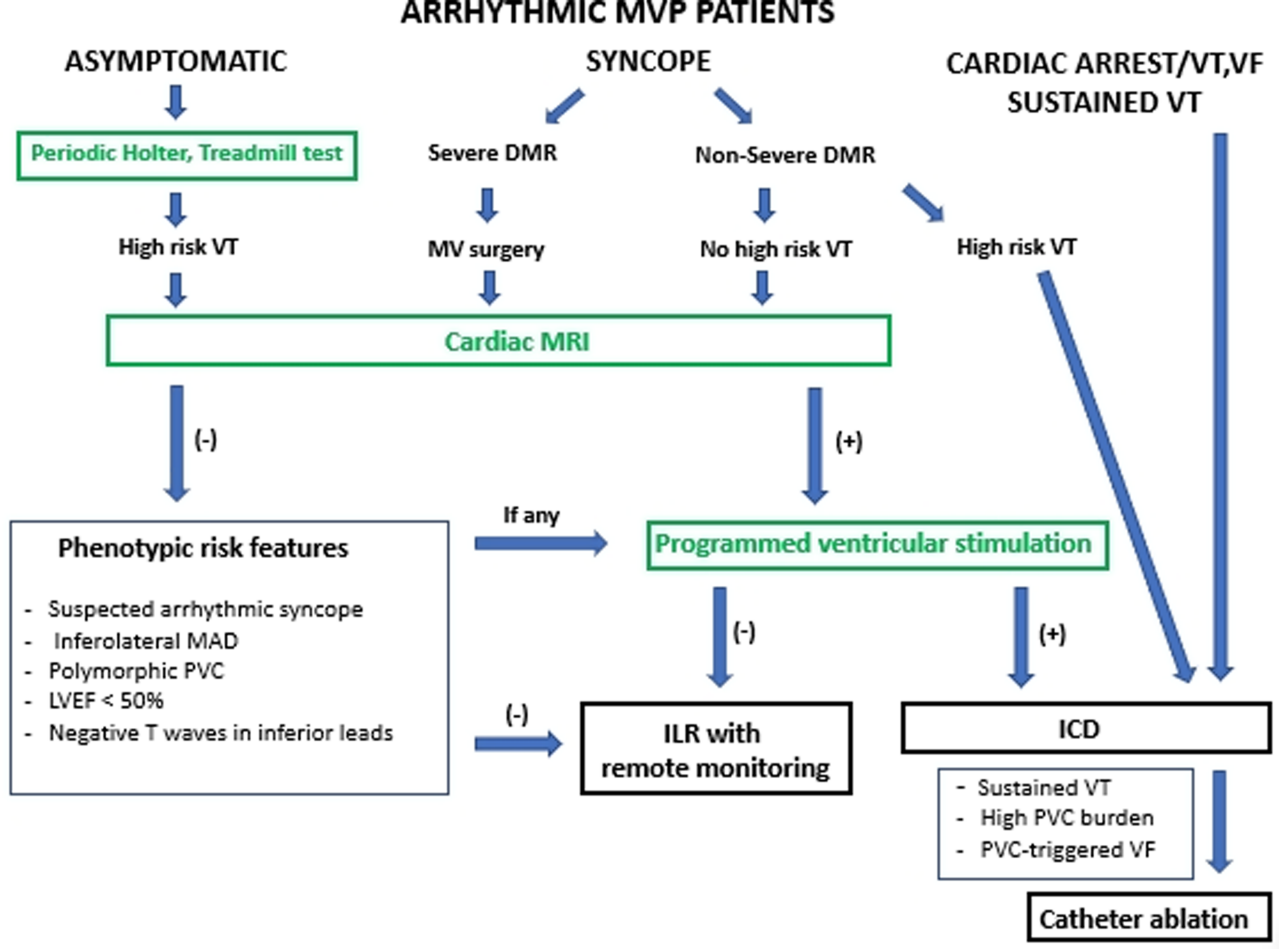
Risk stratification in patients with arrhythmic MVP using clinical and imaging criteria represented by modified EHRA risk stratification scheme (17). Arrhythmic MVP: the presence of MVP with or without MAD, frequent ventricular ectopy (≥5% of total beats), complex ectopy or sustained VAs in the absence of any other well-defined arrhythmic substrate (e.g. active ischemia, ventricular scar due to another defined etiology, primary cardiomyopathy or channelopathy); High risk VT: fast (>180 bpm) non-sustained VT, polymorphic non-sustained VT, Cardiac MRI: only LGE within the mitral apparatus (papillary muscles and peri-annular region) has a clear pathophysiological relevance. DMR, degenerative mitral regurgitation; ICD, implantable cardioverter defibrillator; ILR, implantable loop recorder; LGE, late gadolinium enhancement; LVEF, left ventricular ejection fraction; MAD, mitral annular disjunction; MV, mitral valve; PVC, premature ventricular contraction; VA, ventricular arrhythmia; VF, ventricular fibrillation; VT, ventricular tachycardia.

If MVP is associated with PVCs or non-sustained VT, therapies that prevent SCD and provide symptomatic benefits should be considered. A history of palpitations or syncope or the detection of the phenotypic characteristics of arrhythmic MVP (MAD, leaflet redundancy, and T-wave inversion) should prompt Holter monitoring to identify VA (9, 17). In addition, cardiac MRI should be considered for risk stratification (3, 11). A predictive role of programmed ventricular stimulation to guide therapy for patients with valvular heart disease referred for syncope or VT has been suggested, but uncertainty remains (36). An electrophysiological study is reasonable for patients with syncope if sustained VT is suspected based on symptoms or non-invasive assessment (37). Risk stratification in patients with arrhythmic MVP using clinical and imaging criteria is represented by a modified EHRA statement scheme (Figure 3) (17).

#### ICD therapy

Specific data on ICD therapy for VAs in patients with MVP are lacking. Accordingly, ICD therapy is recommended following general principles of current recommendations for non-ischemic cardiomyopathy (37, 38). Placement of an ICD for secondary prevention of SCD is indicated for patients with MVP and a documented history of sudden cardiac arrest with ventricular fibrillation or sustained VT without reversible causes. For primary prevention, an ICD is recommended in symptomatic heart failure with a LVEF ≤ 35% on optimal medical therapy. Following the recent expert European Heart Rhythm Association (EHRA) consensus statement on arrhythmic MVP and MAD complex, an ICD should be considered for MVP patients with unexplained syncope and high risk VA, defined as non-sustained VT runs ≥180 beats/min detected by ECG or Holter monitoring (17). The option of an ICD may be reasonable for asymptomatic MVP with non-sustained VT and two or more phenotypic risk features (T-wave inversion in inferior leads, repetitive polymorphic PVCs, MAD, redundant MV leaflets, enlarged LA or a LVEF ≤ 50%, and fibrosis within the mitral apparatus by cardiac MRI).

#### Pharmacological treatment

There are no specific data on the efficacy of beta-blockers for the prevention of SCD in the setting of organic MVP, but they are widely used as first-line medical therapy for suppressing frequent symptomatic or complex PVCs and VT. Other anti-arrhythmic agents may also be effective in the management of MVP-associated VAs but must be used with caution, given their potential to cause adverse events. Reduction of VA burden using flecainide combined with beta-blockers after excluding structural heart disease has been recently reported in patients with MVP (39).Class I C sodium-channel blockers should be avoided in cases of MVP with prior myocardial infarction (MI) or hemodynamically significant MR (35).

#### Catheter ablation

In the event of non-responsiveness or contraindication to antiarrhythmic agents, catheter ablation (CA) of symptomatic VAs originating from the PPM in the presence MVP has been reported to be effective (40–44). Ablation for VAs may be considered for patients with appropriate ICD therapies and in cases of PVC-induced cardiomyopathy (37, 38). Catheter stability during the mapping and ablation of MVP-associated VA originating from the papillary muscle may be challenging. Significant progress has been recently made in CA techniques, such as the development of catheters with contact force sensors, which improve safety and allow the creation of larger and deeper lesions. The use of new tools, such as intracardiac echocardiography, which improves catheter positioning on the papillary muscles, or cryoablation catheters that ensure better catheter stability during freezing, has led to higher acute and long-term success rates (43).

A recent study demonstrated the efficacy of CA to reduce the VA burden for patients with MVP and MAD (45). In the presence of MAD, the arrhythmogenic substrate, defined by abnormal local electrograms (low voltage, long-duration, and fractionated, isolated mid-diastolic potentials) on electro-anatomical mapping, was identified in the anterolateral mitral annulus or MAD area (45). An exhaustive list of studies reporting the results of CA for VAs for patients with MVP or MAD is presented in Table 2.

**Table 2:** List of major studies reporting results of catheter ablation of ventricular arrhythmias in patients with MVP.

References	Author	Sample Size	Age (years)	Female (%)	Valvular abnormality (%)	Type of arrhythmia	Common ablation site	Acute success (%)	Mean Follow-up (months)	Follow-up Results
(44)	Syed et al	14	33.8	93	Bileaflet MVP with mild MR	NSVT or sustained VT (57%) History of cardiac arrest, ICD shocks for PVC-triggered VF (43%)	Papillary muscle/fascicular sites (93%) Both LV papillary muscles (55%) Purkinje system (79%)	86	25	Significant decrease in VT burden and appropriate ICD shocks
(41)	Lee et al	9	58.0	78	Bileaflet MVP (89%) < moderate MR (67%)	NSVT (56%)	PPM (48%) Both LV papillary muscles (26%)	60	41	VT recurrences for 25%*
(40)	Bumgarner et al	30	54.3	53	Bileaflet MVP (52%) Posterior MVP (36%) ≥ moderate MR (72%)	PVC (44%) Sustained VT (39%)	Papillary muscle (27%) MV annulus (15%)	67	30	VA recurrences for 26%
(42)	Enriquez et al	25	54.7	64	Bileaflet MVP (72%) Mild to moderate MR (76%)	PVC and NSVT (56%)	PPM (56%) Antero-lateral papillary muscle (32%)	76	31	20% → 6% reduction in PVC burden
(45)	Ezzeddine et al	40	47.0	70	MAD	PVC, sustained VT and PVC-triggered VF	MAD area, anterolateral mitral annulus (substrate ablation)	90	54	9.7% → 4% decrease in PVC burden

*VA recurrence rates reported after single CA procedure.

LV, left ventricle; MAD, mitral annular disjunction; MR, mitral regurgitation; MV, mitral valve; MVP, mitral-valve prolapse; NSVT, non-sustained ventricular tachycardia; PPM, postero-medial papillary muscle; PVC, premature ventricular complex; VA, ventricular arrhythmia; VT, ventricular tachycardia.

Current ESC and AHA/ACC/HRS guidelines for the management of patients with VAs and the prevention of SCD indicate CA for PVCs that trigger recurrent VF as a class I indication (37, 38). CA should be considered after failure of one or more antiarrhythmic agents or according to the patient's preference as a class I (AHA/ACC/HRS) or IIa (ESC) recommendation for symptomatic patients with papillary muscle tachycardia. Regardless of MR severity, frequent PVC and non-sustained VT in the presence of extended phenotypic characteristics of arrhythmic MVP, such as MAD, leaflet redundancy, and T-wave inversion, should lead cardiologists to intensify beta-blocker therapy and discuss CA (35).

#### Knowledge gaps and perspectives

Seminal data suggest that VA are common in patients with MVP but rarely severe and that severe VA is linked to excess mortality. While literature remains sparse and contrasted, recent prospective data provided a definition of the arrhythmic MVP complex, ascertained in an expert consensus to guide future research and clinical trials in order to define the appropriate diagnosis and therapeutic approach.

Holter monitoring is not systematically recommended and the diagnosis of VAs in MVP is still at the discretion of the cardiologist. Arrhythmic MVP is a particular phenotype of MVP that should be part of all MVP comprehensive assessment and trigger episodic or frequent Holter monitoring during follow-up. Little is known about the prevalence and patterns of VAs, in particular transient and subclinical VAs, in mild MVP. Thus, the results and indications of cardiac monitoring by 12-lead Holter monitoring and more recent diagnostic tools, such as sensitive loop recorders, should be prospectively determined in this setting. Establishing the burden, type, sites of origin, and severity of VAs using these methods and exploiting large registries is a starting point for further appropriate prospective research that warrants careful planning. Our understanding of LV remodeling in the setting of MVP at early stages is still insufficient and additional research is needed to elucidate the potential link between VA burden and LV dysfunction.

The severity and prognostic implications of VAs for patients with MVP will require large registries providing sufficient power to define the clinical determinants of outcome. The role of electrophysiological testing and imaging techniques, including cardiac MRI for the detection of ventricular fibrosis and the definition of patterns of the arrhythmogenic substrate, needs to be determined. Further quantification of the identified abnormalities will serve as the basis for establishing data-driven thresholds of clinical severity of arrhythmic MVP.

Pilot data suggest that targeted therapies for MVP-related VAs may be effective, but larger clinical trials will be required to define the indications and optimal timing of CA and its impact on the quality of life and survival. While the risk of life-threatening VA appears to be attenuated after mitral valve surgery, the benefits and indications for ICDs for primary prevention are still poorly defined. More data is needed to identify the burden and character of VAs following valvular surgery using loop recorders and 12-lead Holter monitors.

Despite current knowledge and undeniable recent progress in our understanding of the underlying mechanisms, the association of MVP with VAs is still underestimated. Recent contributions to the arrhythmic MVP complex will set the basis for large and well-defined trials to gather more evidence on genetic, phenotypic, and therapeutic aspects.
